# Recent Advances in Quaternary Ammonium Monomers for Dental Applications

**DOI:** 10.3390/ma17020345

**Published:** 2024-01-10

**Authors:** Xiaoxu Liang, Biao Yu, Liuqi Ye, Danlei Lin, Wen Zhang, Hai-Jing Zhong, Jingwei He

**Affiliations:** 1 Foundation Department, Guangzhou Maritime University, Guangzhou 510725, China; liangxxu@126.com; 2 School of Chemistry and Chemical Engineering, Lingnan Normal University, Zhanjiang 524048, China; y.biao@lingnan.edu.cn; 3 International Cooperative Laboratory of Traditional Chinese Medicine Modernization and Innovative Drug Development of Chinese Ministry of Education (MOE), College of Pharmacy, Jinan University, Guangzhou 510632, China; yeliuqi99@163.com (L.Y.); carolinelin8@163.com (D.L.); 19970042307@163.com (W.Z.); 4 School of Materials Science and Engineering, South China University of Technology, Guangzhou 510641, China

**Keywords:** quaternary ammonium monomers, dental materials, antibacterial activity, dental caries

## Abstract

Resin-based dental materials have been one of the ideal choices among various materials in the treatment of dental caries. However, resin-based dental materials still have some drawbacks, such as the lack of inherent antibacterial activity. Extensive research has been conducted on the use of novel quaternary ammonium monomers (QAMs) to impart antibacterial activity to dental materials. This review provides a comprehensive overview of the recent advances in quaternary ammonium monomers (QAMs) for dental applications. The current progress and limitations of QAMs are discussed based on the evolution of their structures. The functional diversification and enhancement of QAMs are presented. QAMs have the potential to provide long-term antibacterial activity in dental resin composites, thereby prolonging their service life. However, there is a need to balance antibacterial performance with other material properties and the potential impact on the oral microbiome and general health. Finally, the necessity for further scientific progress in the development of novel quaternary ammonium monomers and the optimization of dental resin formulations is emphasized.

## 1. Introduction

Dental caries is generally considered to be the primary cause of dental defects [1,2,3]. To combat dental caries, resin-based dental materials are extensively used in clinical treatment because of their superior cosmetic properties, suitable compressive strength, good biocompatibility, and so on [3,4,5,6]. Nonetheless, dental resins still have several drawbacks, such as a lack of inherent antibacterial activity. Compared to other dental materials, the surface of dental resins is more susceptible to dental plaque accumulation. In addition, the polymerization shrinkage could bring on marginal gaps between the tooth and the material and then potentially lead to secondary caries due to the invasion of pathogenic bacteria in the gaps, while secondary caries may stop the progression of dental resin composites [7,8,9]. Therefore, improving the antibacterial properties of dental resins and extending their service time has attracted considerable academic interest [2,10,11,12].

The conventional method of preparing antibacterial dental resins is the physical blending of low-molecular-weight antibacterial agents such as antibiotics, silver ions, chlorhexidine, and fluoride, with a resin matrix [13,14,15,16,17]. Unfortunately, this method faces several challenges. One of which is the difficulty in uniformly dispersing antibacterial agents in the organic dental resin matrix. In addition, these antibacterial agents have no chemical bonding with the dental resin and tend to exhibit burst-release behavior, which may lead to potential health risks. The corresponding dental materials will rapidly lose their antibacterial activity, resulting in a decline in the mechanical properties of the resins [14,18].

To address these above concerns, dentistry has introduced “immobilized bactericide” into dental materials to achieve enduring antibacterial properties while maintaining mechanical strength [19]. The immobilized bactericide is a monomer that contains both an antibacterial group and carbon–carbon double bonds, which enables the grafting of antibacterial groups onto the polymer networks through chemical bonding. After copolymerization, the antibacterial agent can provide long-term contact antibacterial effects and will not be released from the dental materials, thus avoiding effects on the other properties of the dental materials [20]. Hence, in recent years, investigators have focused on the development of novel antibacterial monomers [21].

**Figure 1:**
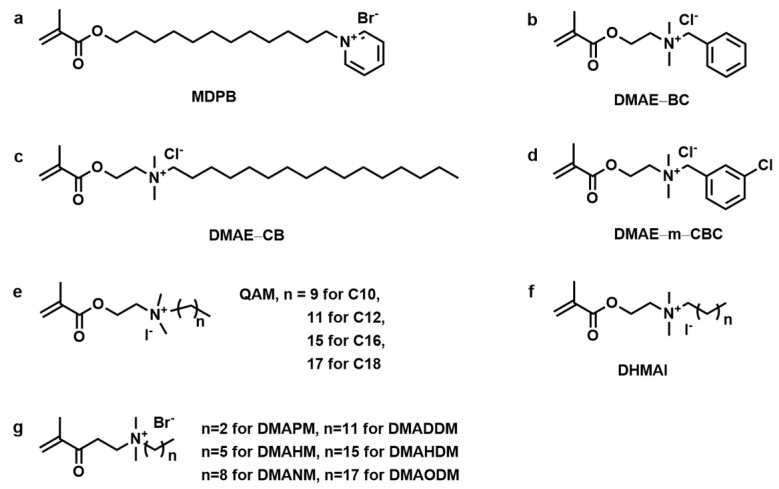
Chemical structures of different mono-methacrylate quaternary ammonium monomers. (**a**) MDPB, (**b**) DMAE–BC, (**c**) DMAE–CB, (**d**) DMAE–m–CBC, (**e**) QAM–n, (**f**) DHMAI, (**g**) QAMs.

Due to their superior antibacterial activity, biocompatibility, and low toxicity, quaternary ammonium salts (QASs) have been extensively employed as antibacterial agents in the pharmaceutical industry, coatings, and water treatment [22]. Polymers with positively charged quaternary ammonium groups have been shown to be effective in reducing bacterial growth in a wide range of applications due to the negatively charged bacterial cell walls. Based on these advantages, Imazato et al. synthesized the first quaternary ammonium monomer used in dentistry, MDPB (1,2-methacryloyloxydodecylpyridinium bromide, as shown in Figure 1a) [19,23]. Taking advantage of the QAS structure, MDPB exhibited remarkable antibacterial activity [24]. Various quaternary ammonium monomers have been synthesized to improve the antibacterial performance and properties of dental resin materials, including low polymerization shrinkage, comparable mechanical properties, and low cost [25]. The structure of quaternary ammonium monomers has also evolved from mono-methacrylate and mono-quaternary ammonium salt to di-methacrylate, bi-quaternary ammonium salt, etc. Quaternary ammonium monomers (QAMs) can also be used in combination with other antibacterial agents to enhance antibacterial properties and further develop into smart monomers and bi-functional monomers. This review summarizes the recent advances in quaternary ammonium monomers for dental applications. The discussion is based on the development of QAM structures and their functions, including antibacterial mechanism, novel chemical structures, smart monomers, radio-opaque, antibacterial bi-functional agents, and the enhancement of antibacterial activity. Table 1 lists the antibacterial properties of representative quaternary ammonium monomers. The conclusions also discuss the future development of these QAMs in dental materials.

## 2. Antibacterial Mechanism of Quaternary Ammonium Monomers

**Figure 2:**
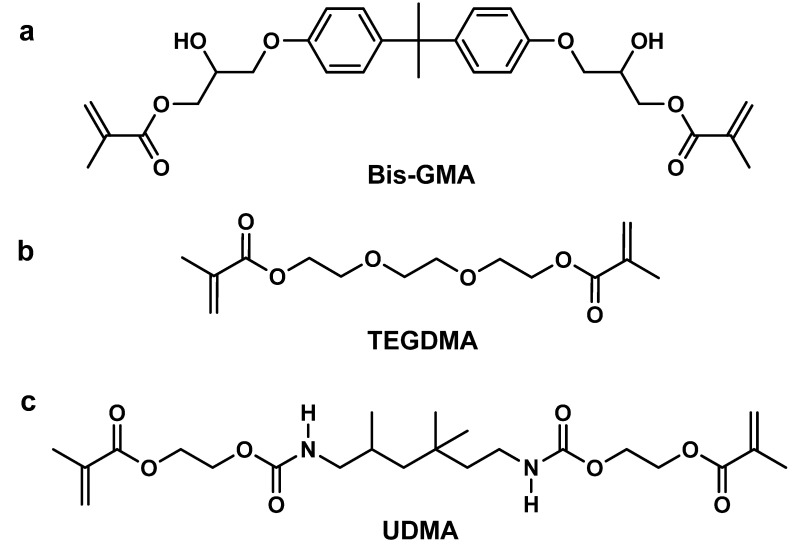
Chemical structures of commonly used monomers in dental resins: (**a**) Bis-GMA, bisphenol A glycerolate dimethacrylate; (**b**) TEGDMA, triethylene glycol dimethacrylate; (**c**) UDMA, rethane-dimethacrylate).

A typical quaternary ammonium monomer comprises polymerizable groups, positively charged quaternary ammonium groups, and an alkyl chain connecting these functional groups. The polymerizable group is generally a (meth)acrylate, which can be co-polymerized with the commonly used dental resin monomers (as shown in Figure 2). The positively charged quaternary ammonium groups as an antibacterial group and is chemically described as NR_4_^+^, where R represents a range of alkyl groups [12,51].

**Figure 3:**
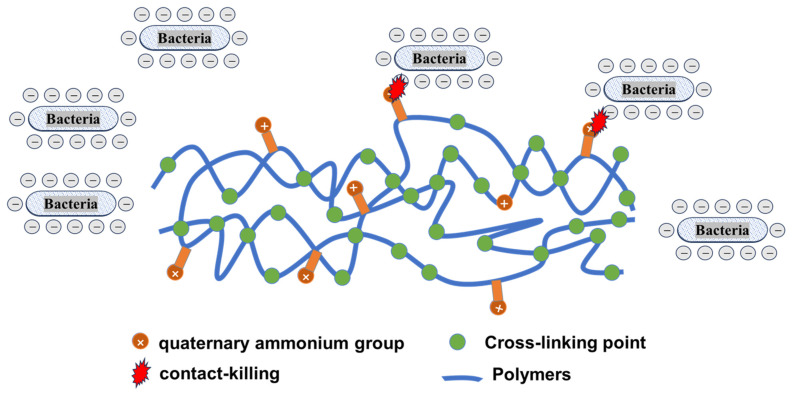
Schematic illustration of the “contact killing” antibacterial mechanism of quaternary ammonium monomers.

The antibacterial mechanism of antimicrobial polymers containing QAM is similar to that of QASs, while the “contact-killing” antibacterial mechanism of QASs is widely recognized (as shown in Figure 3) [51,52,53]. Once bacteria come into contact with the corresponding antimicrobial polymers, they may be destroyed by bursting, and the whole process can be divided into four main steps: The positively charged nitrogen atom in the quaternary ammonium salt group adsorbs the negatively charged bacterial cell wall and cell membrane via electrostatic action. Then, the long alkyl chain penetrates through the cell wall and binds to the cytoplasmic membrane; the integrity of the bacterial cell membranes will be subsequently destroyed, leading to bacterial rupture and death [54,55]. The amphiphilic structure of the QAM is primarily responsible for its antibacterial activity. Many studies have established that increasing the length of the alkyl side chain promotes step (3) and (4) and that the antibacterial activity of QAM incorporated into dental polymers is reinforced by increasing the length of the alkyl side chain [38,54,55,56,57,58]. Nevertheless, as the chain length increases, a critical point (threshold value) is reached, beyond which the antimicrobial activity decreases. The optimal chain length or threshold point for a given quaternary ammonium methacrylate (QAM) is determined by the final polymer structures and networks, such as hydrophobicity and cross-linking density of the prepared polymer [30,38,55,59].

## 3. Mono-Methacrylate Quaternary Ammonium Monomers

MDPB is the first reported quaternary ammonium monomer used as an antibacterial agent in dental materials. The antibacterial activity and cytotoxicity of MDPB as well as the relevant polymers have been widely investigated [26,60,61]. MDPB displayed strong bactericidal activity against seven streptococci, with MIC (minimum inhibitory concentrations) and MBC (minimum bactericidal concentrations) ranging from 3.13 to to 25.0 μg/mL and 6.25 to 62.5 μg/mL, respectively [26]. Time-kill determination showed a rapid killing effect of MDPB at 250 μg/mL or above, and all cells were killed within 1 min by MDPB at 500 μg/mL or above [60,62].

MDPB has been successfully integrated into the Clearfil Protect Bond Primer, a commercial dental bonding system comprising a two-step self-etching dental bonding system that contains 5% MDPB primer. The primer exhibits strong antibacterial activity against *S. mutans* (*Streptococcus mutans*), *L. casei* (*Lactobacillus casei*), and *A. naeslundii (Actinomyces naeslundii)*. It addresses the challenge of residual bacteria in cavities, contributing to better prognoses for minimal restorative treatments of dental caries. Additionally, the MIC/MBC results indicate the primer’s efficacy in inhibiting and killing the tested bacterial species. This attribute provides a promising advantage for clinical applications [27].

After the introduction of MDPB, the development and utilization of quaternary ammonium monomers in resin-based dental materials have been extensively studied. Xiao et al. successfully synthesized three mono-methacrylate quaternary ammonium monomers as shown in Figure 1b–d: DMEA-BC (methacryloxylethyl cetyl ammonium chloride), DMAE-CB (methacryloxylethyl benzyl dimethyl ammonium chloride), and DMAE-m-CBC (methacryloxylethyl m-chloro benzyldimethyl ammonium chloride) [28,63]. The study found that all tested bacterial strains were exposed to the three monomers, with DMAE-CB exhibiting the lowest MIC, ranging from 1.2 to 4.8 μg/mL. The killing rate against *S. mutans* of DMAE-CB reached 100% after 10 min. The antibacterial activity of DMAE-CB was found to be similar to that of chlorhexidine, a gold standard for antibacterial application, indicating its potential as an effective alternative [28]. The antibacterial efficacy of the adhesive containing Single Bond 2 and different percentages of DMAE-CB (1%, 2%, and 3%) was evaluated, respectively. The adhesives that incorporated 3% DMAE-CB showed the best antibacterial activity, and this effect lasted for at least 180 days. Furthermore, the cured specimens’ surface inhibited the growth of *S. mutans*. The introduction of DMAE-CB had no significant effect on the bonding performance of these experimental adhesives. This research indicated that DMAE-CB offers the opportunity to serve as an efficient antibacterial monomer for dental adhesives [29].

He et al. synthesized a series of antibacterial quaternary ammonium methacrylate monomers (QAM-n, quaternary ammonium methacrylate, as shown in Figure 1e) with varying substituted alkyl chain lengths ranging from 10 to 18. Next, 5 wt% QAM was incorporated into bisphenol A glycerolate dimethacrylate/triethyleneglycol dimethacrylate (Bis-GMA/TEGDMA) dental resin as an immobilized antibacterial agent. The study revealed that the length of the alkyl chain of QAMs had a notable effect on the antibacterial activity of the copolymer. Copolymers containing C18 and C16 exhibited the highest inhibitory efficacy on both young and mature biofilms. In addition, the study found that incorporating 5 wt% QAMs into the dental resin had minimal negative impact on double bond conversion, flexural strength, and modulus. However, QAM-n is not readily miscible with dental resin at doses exceeding 5 wt%, which could considerably restrict the antibacterial effectiveness of the test resins [30]. Overall, this finding suggests that the antibacterial properties can be tailored based on the specific QAMs used, providing a customizable approach to addressing bacterial accumulation in dental materials. The results of the study are significant in light of the potential implications for clinical practice in the field of restorative dentistry.

Cherchali et al. investigated the effectiveness of an antibacterial dental composite containing DHMAI (dimethyl-hexadecyl-methacryloxyethyl-ammonium iodide, as shown in Figure 1f) [31,64]. Incorporating DHMAI enhanced the composite’s antibacterial activity. The composite with 7.5% DHMAI achieved a reduction of ~98% in colony-forming unit (CFU) and ~50% of metabolic activity. It also exhibited acceptable mechanical properties, improved thermal behavior (glassing transition temperature and thermal degradation), and a higher conversion degree [31]. After 3 months of aging, the DHMAI-containing composition demonstrated better structural stability and resistance against biodegradation compared to the control composite, likely due to the incorporation of DHMAI [64]. Nonetheless, when the DHMAI content reached 10%, the flexural strength (*FS*) and flexural modulus (*FM*) of the composites decreased significantly, although they remained within the *FS* limit of standard ISO 4049 [65]. These findings have implications for improving the longevity and antibacterial properties of dental restorations, offering potential benefits for clinical applications in restorative dentistry.

Zhou et al. synthesized a series of QAMs with alkyl chains ranging from 3 to 18 carbons (DMAPM = dimethylaminopropyl methacrylate; DMAHM = dimethylaminohexyl methacrylate; DMANM = dimethylaminononyl methacrylate; DMADDM = dimethylaminododecyl methacrylate; DMAHDM = dimethylaminohexadecyl methacrylate; DMAODM = dimethylaminooctadecyl methacrylate, as shown in Figure 1g) and investigated the relationship between chain length, quaternary amine charge density, and antibacterial activity of these monomers [32]. The antibacterial activity of the bonding agent containing QAMs increased initially with longer alkyl chains but decreased thereafter. Conversely, the bonding agent containing DMAHDM with an alkyl chain length of 16 carbon displayed the best antibacterial activity [32]. The result could be ascribed to a longer alkyl chain length augmenting the ability of quaternary ammonium to penetrate bacterial membranes, leading to physical disruption [32,66]. The relationship between charge density and antibacterial activity was also investigated. Increasing the quaternary ammonium monomer dosage raised the charge density of dental resin. The results indicated that stronger antibacterial activity occurred as charge density increased. Moreover, a higher charge density significantly reduced the early attachment of bacteria, resulting in a 4 *log* decrease in biofilm colony-forming units (CFUs) compared to the commercial control [32,39]. This work introduces innovative approaches to developing antibacterial bonding agents and provides insights into the potential use of tailored materials to combat dental plaque and caries.

In recent years, DMADDM and DMAHDM have become the most commonly used quaternary ammonium mono-methacrylate monomers due to their facial synthesis and strong antibacterial activity, as reported in numerous studies [51,67,68,69,70,71,72,73]. Wang et al. evaluated the antibacterial activity of a bioactive dental composite containing 3% DMAHDM against *S. mutans*, *S. sanguinis* (*Streptococcus sanguinis*), and *S. gordonii* (*Streptococcus gordonii*) in a biofilm model. The experimental groups showed a 2–3 log reduction in CFUs compared to the control groups. DMAHDM-containing composite resin significantly inhibited an early attachment of bacteria at 4 h and early biofilm formation at 24 h [33]. However, the resin composites containing 5 wt% DMAHDM had a lower FS than the control group [69,74].

*Nevertheless,* despite their potential benefits, the use of these mono-methacrylate monomers is still limited by their long alkyl chains and the fact that they only have one polymerizable group in their molecular structure. Additionally, these monomers do not easily mix in high concentration with commonly used commercial di-methacrylate monomers, like Bis-GMA, TEGDMA, and UDMA (urethane dimethacrylate) [10,21]. Moreover, high concentrations of mono-methacrylate monomers have a significant negative effect on the network structure and mechanical strength of dental resins [26,38,75]. Therefore, to achieve comprehensive performance, dental resins should contain a lower amount of mono-methacrylate monomer in dental resins. For example, the maximum amount of MDPB in dental resins should not exceed 0.4% [35]. Further modification of the quaternary ammonium monomer is necessary.

## 4. Di-Methacrylate Quaternary Ammonium Monomer

To address the issues with mono-methacrylate quaternary ammonium monomers, using di-methacrylate quaternary ammonium monomers that have two polymerizable groups seems to be a better choice for an immobilized antibacterial agent in dental resins. Consequently, various types of di-methacrylate quaternary ammonium monomers have been developed, and their application in dental resin materials has been investigated [34,40].

**Figure 4:**
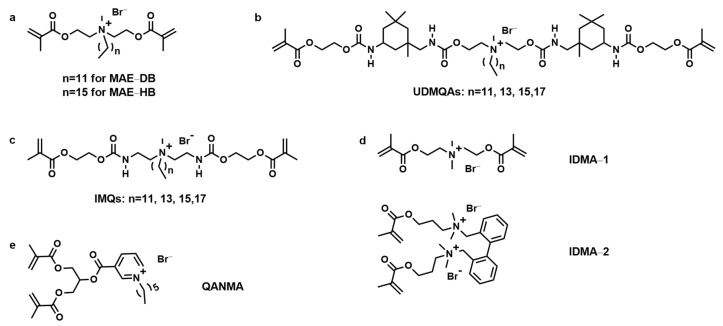
Chemical structures of different di-methacrylate quaternary ammonium monomers. (**a**) MAE–DB and MAE–HB, (**b**) UDMQAs, (**c**) IMQs, (**d**) IDMA–1 and IDMA–2, (**e**) QANMA.

Huang and colleagues synthesized di-methacrylate quaternary ammonium monomers: MAE-DB and MAE-HB (2-methacryloxylethyl dodecyl methyl ammonium bromide and 2-methacryloxylethyl hexadecyl methyl ammonium bromide, as shown in Figure 4a). They also evaluated the monomers’ antibacterial activity and cytotoxicity [34]. Both MAE-DB and MAE-HB demonstrated strong antibacterial activity against eight strains of oral bacteria, with MBC values ranging from 12.2 to 24.4 μg/mL and 6.2 to 48.8 μg/mL, respectively. The median lethal concentration of these monomers exhibited a lower cytotoxicity than Bis-GMA on human gingival fibroblasts, with a range of 10~20 μg/mL [34]. Unlike previous quaternary ammonium monomers, MAE-DB can be incorporated into dental resins at a maximum of 10% by mass, with the antibacterial activity lasting up to 180 days. By reducing *gtfB* expression and impairing membrane integrity, the polymers containing MAE-DB and MAE-HB exhibited durable antibacterial effects against *S. mutans*. The study suggests that the incorporation of these monomers into resin materials can play an important role in preventing the occurrence of secondary caries, a common issue with dental restorations [35].

Liang et al. synthesized four of di-methacrylate quaternary ammonium monomers, UDMQAs, with alkyl chains ranging from 12–18 (urethane dimethacrylates quaternary ammonium methacrylate, as shown in Figure 4b). They studied the application of UDMQAs in dental resins and found them to be miscible with TEGDMA in a high ratio of 50 wt%:50 wt%. Due to their quaternary ammonium structure and high molecular weight, all UDMQA-based polymers showed antibacterial activity and reduced polymerization shrinkage compared to Bis-GMA-based polymers. However, UDMQA-based polymers had lower *FS* and *FM* and higher water sorption and sol fraction [36]. UDMQA-12 had no negative influence on the degree of double bond conversion, and it also reduced the polymerization shrinkage rate of the resin, which are very important in their application in dental resins. As for the UDMQA-12-containing polymers, they displayed a significant antibacterial activity at a concentration of 30% or more. However, the incorporation of UDMQA-12 resulted in a reduction in *FS* and *FM* of the resulting polymers, which persisted with increasing UDMQA-12 [37]. The antibacterial activity and reduced polymerization shrinkage of UDMQAs are significant benefits that may outweigh their drawbacks in specific clinical scenarios. This work suggests their potential application use as a base compound in dental resins.

Another series of di-methacrylate quaternary ammonium monomers with alkyl chains ranging from 12 to 18, IMQs, were also synthesized by Liang et al. (quaternary ammonium dimethacrylate monomers, as shown in Figure 4c). These IMQs were highly miscible with Bis-GMA/TEGDMA. When introduced into dental resin at a concentration of 10% or more by weight, the dental resin that contained IMQs showed antibacterial activity. The polymers that contained IMQ-16 displayed the best comprehensive performance, and the ideal concentration of IMQ-16 ranged 5–10 wt%, balancing both antibacterial activity and mechanical properties [38]. This work provides valuable insights into the influence of alkyl chain length on antibacterial activity, highlighting the importance of alkyl chain length in designing effective antibacterial monomers.

Antonucci et al. developed two new di-methacrylate quaternary ammonium monomers, IDMA-1 and IDMA-2 (bis(2-methacryloyloxyethyl)dimethylammonium bromide, and 2,2′-bis(methacryloxyloxyethyl dimethylammonium bromide-1,1′-benzyl) as shown in Figure 4d), to create low-viscosity ionic antibacterial monomers that could be compatible with existing di-methacrylate-based dental monomers [39]. IDMA-1 was found to be miscible with the Bis-GMA/TEGDMA resin system, and its potential use in dental resins was investigated. Polymers containing 10% or more IDMA-1 can significantly reduce bacterial growth without compromising mammalian cell viability, indicating their potential application in antibacterial dental materials. However, those polymers containing ≥20% IDMA-1 present considerable cytotoxicity toward macrophages. Therefore, achieving a balance between antibacterial activity and cytotoxicity is essential for their practical application [39]. Overall, IDMA-1 could contribute to the development of improved biomaterials with reduced bacterial adhesion, addressing a critical need in the field of dental materials.

To improve the biocompatibility and antibacterial activity dental resins, Li et al. synthesized QANMA (1,3-bis(methacryloyloxy)propyl-carbonyl-hexylpyridinium bromide, as shown in Figure 4e), a series of novel quaternized pyridine di-methacrylate monomers based on niacin and incorporated them into dental resins [40]. The results indicated that QANMA significantly endows the dental resin with antibacterial properties, particularly at concentrations of 10% and 20%. The incorporation of QANMA also reduces the polymerization shrinkage of the resins, which is beneficial for minimizing the risk of micro-leakage and secondary caries. Nevertheless, the higher concentration of QANMA led to increased water sorption and solubility, as well as weaker mechanical strength. Despite these drawbacks, the *FS* of these resins still meet the requirements of the ISO standard [40]. The study highlights the QANMA’s potential as an antibacterial agent in dental materials, with emphasis on the need for further optimization and biocompatibility evaluation.

Beneficial for their structure, the di-methacrylate structure can significantly improve their miscibility in dental resins and increase the cross-linking density of the resin while reducing the dissolution of unreacted monomers. This improvement is advantageous for enhancing overall performance of the polymers [21,76]. These monomers can also can the polymers with long-lasting antibacterial activity. However, di-methacrylate monomers have a lower concentration of quaternary ammonium salts compared to the mono-methacrylate quaternary ammonium monomers. As a result, a higher dosage of di-methacrylate quaternary ammonium monomers is required in the resin to achieve the desired antibacterial performances. On the other hand, an excessive concentration of quaternary ammonium monomers can greatly reduce the mechanical properties of the resin due to the hydrophilic structure of the quaternary ammonium salt, rendering it unsuitable for clinical application [23,36,77,78]. It is crucial to achieve a balance between the antibacterial properties and mechanical strength in the application of these monomers. 

## 5. Bi-Quaternary Ammonium Monomers

The antibacterial properties of antibacterial dental materials depend primarily on the concentration of quaternary ammonium salt groups in the polymers. Therefore, introducing monomers that contain multiple quaternary ammonium salt groups as antibacterial agents is expected to enhance antibacterial activity while minimizing the use of such monomers [79,80].

**Figure 5:**
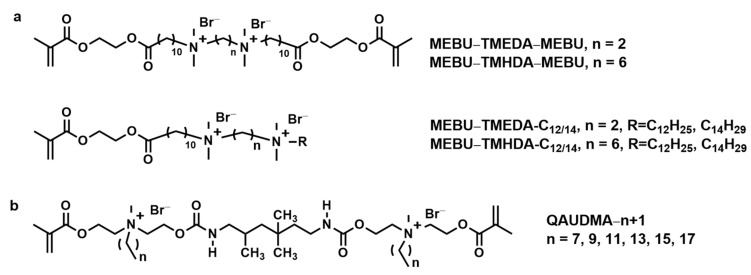
Chemical structures of (**a**) Gemini QAS and (**b**) QAUDMAs.

Qiu et al. synthesized two series of bi-quaternary ammonium monomers: Gemini quaternary ammonium salts (Gemini QAS, as shown in Figure 5a) [41]. One series consisted of symmetrical Gemini QASs with two di-methacrylate groups: N,N,N′,N′-tetramethyl-dimethylene-bis(1-undecanoate-2-(2-methacryloyl-oxy) ethyl) bromide (MEBU-TMEDA-MEBU), N,N,N′,N′-tetramethyl-hexamthy-lene-bis(1-undecanoate-2-(2-methacryloyloxy) ethyl) bromide (MEBU-TMHDA-MEBU), while the other series consisted of asymmetric Gemini QASs with only one methacrylate group: N,N,N′,N′-tetramethyl-dimethylene-dodecyl-1-undecanoate-2-(2-methacry-loyloxy)ethyl) bromide (MEBU-TMEDA-C_12_), N,N,N′,N′-tetramethyl-dimethylene-tetradecyl-1-undecanoate-2-(2-methacry-loyloxy)ethyl) bromide (MEBU-TMEDA-C_14_), N,N,N′,N′-tetramethyl-hexamthylene-dodecyl-1-undecanoate-2-(2-methacryloyl-oxy)ethyl) bromide (MEBU-TMHDA-C_12_), and N,N,N′,N′-tetramethyl-hexamthylene-tetradecyl-1-undecanoate-2-(2-methacryloyloxy) ethyl) bromide (MEBU-TMHDA-C_14_). Additionally, a long spacer was specifically designed to connect the methacrylate group and the cationic group in the structure of these monomers, which facilitates enhancing the antibacterial activity of the dental resins. Six of these monomers were incorporated into a dental restoration resin system. Among these, four monomers showed superior antibacterial activity against *Staphylococcus aureus (S. aureus)* with MIC values below 10 μg/mL, compared to conventional antibacterial agents. On the other hand, the symmetric Gemini QAS monomers exhibited weaker antibacterial properties than the asymmetric Gemini QAS. It could be attributed to the molecule’s symmetrical structure, which fixes both ends of the molecule and reduces the availability of the free quaternary ammonium group [41]. These results suggest a tailored design strategy in creating dental materials with antibacterial properties.

Chrószcz et al. developed a series of novel bi-quaternary ammonium urethane-dimethacrylate monomers with alkyl chains of 8~18 carbon atoms, QAUDMAs (quaternary ammonium urethane-dimethacrylate, as shown in Figure 5b). Their potential application in antibacterial dental composites has been investigated [81,82]. The QA: TEGs resins containing 60% QAUDMA and 40% TEGDMA by mass exhibited significant antibacterial activity against both *S. aureus* and *Escherichia coli* (*E. coli*), in addition to lower polymerization shrinkage and a higher degree of double bond conversion [42]. However, QA: TEGs resins exhibited weaker mechanical strength (*FS* = 37.37 − 20.13 MPa, *FM* = 459.4 − 851.6 MPa) and higher water sorption and solubility when compared to the BG:TEG control group. These results may be attributed to the influence of the length of the N-alkyl substituent. Therefore, the results indicated that these composites were unsuitable for the application in dental materials and require further modification [82]. 

**Figure 6:**
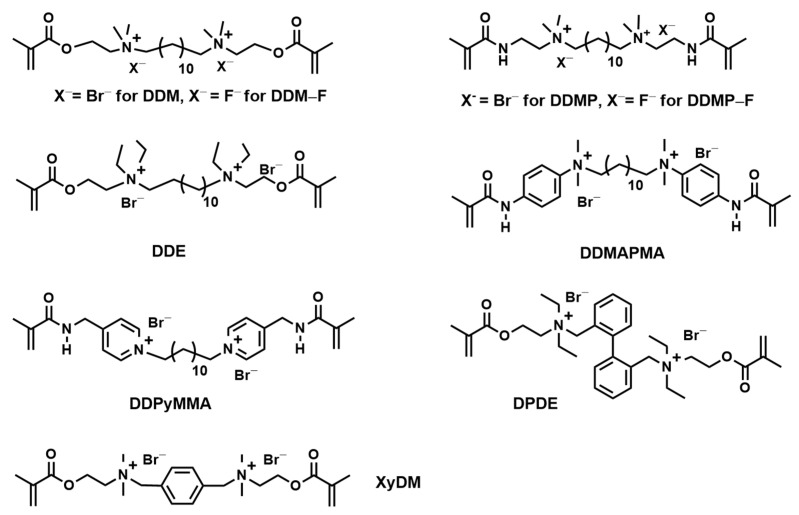
Chemical structures of bis-QAMs.

Fanfoni et al. synthesized nine antibacterial di-methacrylate monomers based on bis-quaternary ammonium salts (bis-quaternary ammonium di-methacrylates named bis-QAMs, as shown in Figure 6) [62]. Compared to MDPB, two of bis-QAMs exhibited a comparable and even stronger antibacterial activity against *S. mutans* [62]. Moreover, the antibacterial activity of the monomer is related to the flexibility of the group between the two quaternary ammonium salts, the balance of hydrophilicity and hydrophobicity of the monomer as well as the steric hindrance of the quaternary ammonium salt side group, which are important in the design of novel antibacterial monomer. This study demonstrated that the newly synthesized monomers, such as N,N′-bis[2-((methacryloyl)oxy)ethyl]-N,N,N′,N′-tetramethyl-N,N′-dodecyl diammonium bromide (DDM), N,N′-bis[2-((methacryloyl)oxy)ethyl]-N,N,N′,N′-tetraethyl-N,N′-dodecyl diammonium bromide (DDE), N,N′-bis((4-methacryloyl)amino)phenyl-N,N,N′,N′-tetramethyl-N,N′-dodecyl diammonium bromide (DDMAPMA), and bis[4-(((2-methylacryloyl)amino)methyl)-1,12-dodecyl dipyridinium bromide (DDPyMMA), have the potential to be incorporated into dental restorative materials to provide long-lasting antibacterial properties [62].

**Figure 7:**
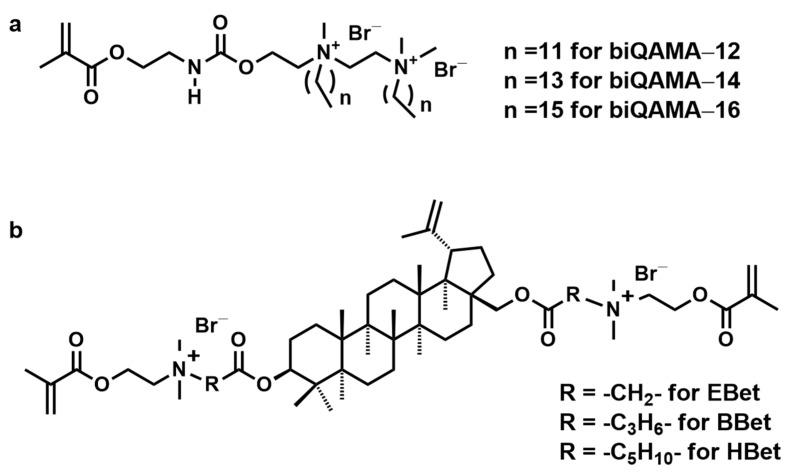
Chemical structures of (**a**) biQAMAs and (**b**) Bis-QADM-Bets.

He et al. synthesized a series of novel bi-quaternary ammonium monomers, biQAMAs (bi-quaternary ammonium methacrylates, as shown in Figure 7a) and evaluated their effectiveness as antibacterial agents in dental resin composites (DRCs) [43]. The DRCs that contained 5 wt% biQAMAs displayed considerable antibacterial effects, with an antibacterial rate exceeding 90% against *S. mutans*. The DRCs had comparable physicochemical properties, while also meeting the ISO standard requirement despite an increase in water sorption (*WS*). Among the DRCs, biQAMA-12 showed an exceeding 99% antibacterial efficiency and had no negative effect on the cytotoxicity of DRCs. These beneficial properties can be ascribed to the dodecane alkyl chain and higher surface charge. The findings indicated the potential of these materials for combating bacterial growth in dental applications. Meanwhile, biQAMA-12 is present as the optimal antibacterial agent for antibacterial DRCs [43].

Zhang et al. developed three types of bis-quaternary ammonium di-methacrylate monomers, Bis-QADM-Bet (di(dimethylaminoethyl methacrylate ethylammonium bromide)betulin-based derivative, abbreviated as EBet; di(dimethylaminoethyl methacrylate butylammonium bromide)betulin-based derivative, abbreviated as BBet; and di(dimethylaminoethyl methacrylate hexylammonium bromide)betulin-based derivative, abbreviated as HBet, as shown in Figure 7b) based on the natural product botulin and copolymerized them with Bis-GMA/TEGDMA at a concentration of 10 wt% [44]. The experimental resins have stronger antibacterial activity with a more than 99.9% antibacterial efficiency against *S. mutans*. Among these resins, the 1BBet4B5T (10 wt% BBet, 40 wt% Bis-GMA, and 50 wt% TEGDMA) resin with a median alkyl chain length exhibited the best performance. The 1BBet4B5T resin had acceptable mechanical strength and polymerization behavior and the strongest antibacterial properties, which could significantly inhibit the growth of *S. mutans.* Moreover, this work provides insight into the potential antibacterial mechanism of contact-killing through the following steps: an electrostatic attraction and leakage of cell contents; then, dental resins containing Bis-QADM-Bet inhibit the expression of all the targeted genes (*gtfB*, *gtfC*, and *gtfD*) of *S. mutans*, disrupt the biofilm metabolism, and ultimately lead to bacterial death. This work provides a promising strategy for the development of natural product-based antibacterial dental resins as well as the exploration of more accurate antibacterial mechanisms [44].

In summary, these bi-quaternary ammonium monomers showed promise for future developments of quaternary ammonium-based antibacterial dental materials.

## 6. Quaternary Ammonium Monomers as Radio-Opaque and Antibacterial Bifunctional Agents

Non-destructive techniques are typically used to assess the quality of dental treatment, making iodine-containing compounds valuable for improving the visibility of dental treatments. Iodine is highly radio-opaque due to its high electronic density and is widely used as a radioactive medium in modern medicine [83,84,85]. As a result, quaternary ammonium monomers, in addition to having strong antibacterial activity, can impart radio-opaque properties to dental materials by using iodine as a counter anion [46,83].

**Figure 8:**
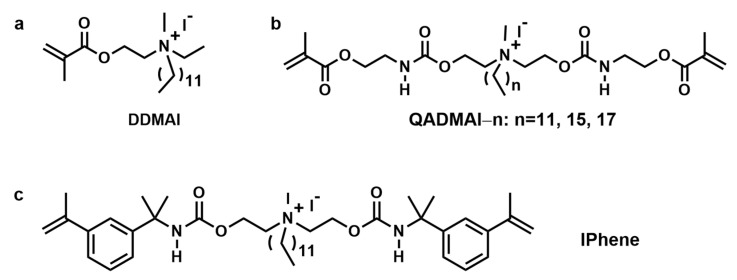
Chemical structures of different radio-opaque and antibacterial bi-functional agents. (**a**) DDMAI, (**b**) QADMAI–n, (**c**) IPhene.

Using iodine anion as counter anion, He et al. designed and synthesized several novel quaternary ammonium monomers with iodine anion (2-Dimethyl-2-dodecyl-1-methacryloxyethyl ammonium iodine, abbreviated as DDMAI; N,N-bis[2-(3-(methacryloyloxy)propanamido)ethyl]-N-methylalkyl ammonium iodide, abbreviated as QADMAI; polymerizable quaternary ammonium monomer with iodine anion, abbreviated as IPhene, as shown in Figure 8). These bifunctional monomers were then copolymerized with Bis-GMA/TEGDMA dental resin to prepare antibacterial and radio-opaque dental materials [45,46,47,48,49]. The results demonstrated that the increasing concentration of bifunctional monomers enhanced the antibacterial performance and radio-opaque properties of the relevant polymers. Unfortunately, the radio-opaque properties of the polymers could not be improved to a high level due to the low total concentration of iodine. It is difficult to make the balance between radio-opaque properties, mechanical strength, and antibacterial activity. For example, incorporating IPhene into the dental resin led to a decrease in flexural strength and modulus, as well as double bond conversion, particularly at higher IPhene concentrations. The dental resin containing IPhene showed both antibacterial activity and radio-opacity at IPhene concentrations of 30 wt% and 40 wt%. However, the *FS* and *FM* of these polymers are lower than those of the control group, although they still meet the requirements of the ISO standards [49]. Therefore, further optimization of bifunctional monomer structure and resin formulation is required [45,48,49].

## 7. Quaternary Ammonium Monomers as Smart Monomers

Biofilms are widely considered to be the initiating factor of secondary caries [13]. Dental resin composites containing QAMs have shown a long-term antibacterial activity. However, such antibacterial agents will kill all the bacteria on the surface of the composites and disturb the balance of the oral microbiome, which may be unfriendly to overall health [86]. In addition, the acid generated by the acidogenic bacteria causes the pH to drop below 5.5 during the development of caries [87,88]. Therefore, the “smart” monomer with reversible pH response will be the ideal antibacterial agent for dental resin composites [50,89].

**Figure 9:**
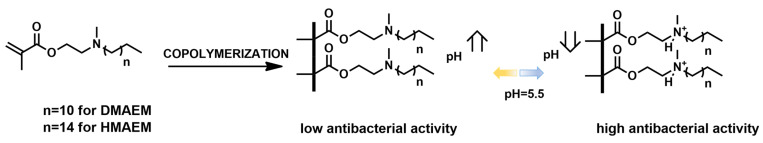
Chemical structures and schematic illustration of antibacterial mechanism of DMAEM/HMAEM.

Liang et al. developed, designed, and synthesized two “smart” tertiary amine group-containing monomers, TAs (as shown in Figure 9)—DMAEM (dodecylmethylaminoethyl methacrylate) and HMAEM (hexadecylmethylaminoethyl methacrylate)—which can be protonated to form quaternary ammonium salts at low pH, and be deprotonated at high pH [50]. The result indicated that DMAEM and HMAEM have antibacterial activity only in an acidic environment. The incorporation of DMAEM and HMAEM had no negative effect on the mechanical properties and biocompatibility of the tertiary amine (TA)-modified resin adhesives (TA@RAs) [50]. Due to the reversible pH response of TAs, TA@RAs showed a long-term antibacterial activity and the ability to prevent secondary dental caries. Additional research was conducted to investigate the ability of resin infiltrate-containing DMAEM to prevent white spot lesions (*WSLs*). The incorporation of DMAEM was successful in preventing *WSLs*, stopping early caries, and protecting enamel hardness [89]. The novel smart monomers are highly promising for application in antibacterial dental resin composites.

## 8. Antibacterial Property Enhancement of Dental Resins

Although quaternary ammonium monomers can provide significant antibacterial effects to dental resin composites, studies have explored the combination of these monomers with other antibacterial agents to improve the overall performance of a variety of dental resin composites. Numerous studies have demonstrated the effectiveness of this approach [70,71,72].

Fluorine is widely recognized as an element with anti-caries and antibacterial properties. It can also inhibit the formation of caries by interfering with the plaque formation and inhibiting the growth and metabolism of bacteria [90,91]. Mitwalli et al. fabricated a novel series of nanocomposites containing nano-calcium fluoride (nCaF_2_) and DMAHDM, which could endow the composites with both remineralizing and antibacterial properties. Compared with the control composites, the nCaF_2_-DMAHDM composites showed comparable mechanical properties and a higher conversion degree, meeting ISO recommendations. The high release of F^−^ and Ca^2+^ provided the potential for remineralization, without compromising their mechanical properties [71]. In addition, nCaF_2_-DMAHDM composites significantly reduced biofilms formation and lactic acid production, with a 3~4 log reduction in CFU, which is crucial for promoting long-term sustainability of restorations. The results suggest that recurrent caries could be prevented by nanocomposites containing nCaF_2_ and DMAHDM, indicating their potential for use in dental restorations.

**Figure 10:**
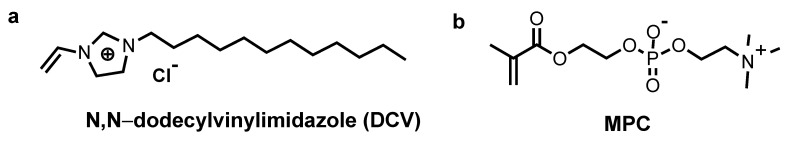
Chemical structures of (**a**) DCV and (**b**) MPC.

Zhang et al. fabricated a series of dental resin adhesives containing NaF nanoparticles and imidazole-based *quaternary ammonium monomers* DCV (N,N-dodecylvinylimidazole, as shown in Figure 10a) [92]. The initial release rate of fluoride ion was decreased by the electronic interaction between F^−^ and N^+^ and by the hydrophilicity of DCV. The adhesive obtained dual antibacterial mechanisms due to the co-incorporation of fluoride ion and the quaternary ammonium group. This dual mechanism enhances the overall antibacterial activity of the adhesive. In addition, the C=C double conversion rate was not adversely affected by DCV, but the hydrophilicity and bonding strength of the corresponding adhesives were. Consequently, the adhesive formulation needs to be further optimized. This work highlights the potential clinical significance of incorporating NaF nanoparticles and DCV into dental resin adhesives for efficient dental restoration and the prevention of secondary caries, and demonstrates the promising strategy in the development of antibacterial dental resin adhesives [92].

Direct contact between bacteria and dental resin is considered a fundamental requirement for the antibacterial activity of quaternary ammonium monomers. It is mainly dependent on the direct contact between bacteria and dental resin. However, the hydrophobic surface of dental resins could be covered with salivary protein, and the antibacterial effect in the oral cavity will be significantly reduced as a result [93]. Due to its super hydrophilic property and the presence of water structures on its surface, the coating containing MPC (2-methacryloyloxyethyl phosphorylcholine, as shown in Figure 10b) could reduce the adsorption of proteins and inhibit the adherence of streptococcal [94,95]. Several studies have been carried out on the antibacterial performances of dental resin composites combining quaternary ammonium monomers and MPC as results. Zhang et al. developed a series of dental composites containing 3% MPC and 1.5% DMAHDM, which have strong protein-repellent and antibacterial activity. Biofilm growth and lactic acid production were significantly reduced on these composites, while biofilm CFU counts on composite with 3% MPC + 1.5% DMAHDM were more than three orders of magnitude lower than that of commercial control. In addition, mechanical properties were not compromised [96]. Pasiree Thongthai et al. reported a novel copolymer by combining MDPB, an antibacterial component, with MPC, a polymer with protein-repellent properties. The resulting copolymer coating demonstrated enhanced hydrophilicity, reduced protein adsorption, and an effective killing of *S. mutans* [97]. These innovative approaches presented a promising solution for developing dental materials with improved antibacterial and protein-repellent properties [96,97].

## 9. Conclusions

This article reviews the historical evolution of quaternary ammonium monomers (QAMs) based on their structure. It discusses the limitations of current works, and the functional diversification and enhancement of QAMs. Evidence suggests that incorporating quaternary ammonium monomers is a promising path to developing dental resin composites with a long-term antibacterial activity, which can prolong their service life of dental resin composites. However, the high efficacy of quaternary ammonium salts in reducing bacterial adhesive and biofilm formation must be weighed against their potential negative effects on other dental material performances, such as mechanical strength, biocompatibility, and water absorption. Additionally, the use of these antibacterial agents has the potential to disrupt the balance of the oral microbiome, which may have negative impacts to general health. More attention should be paid to the long-term safety of such monomers. Therefore, to achieve long-lasting antibacterial activity, smart functionality, biosafety, and a balance between antibacterial performance and comprehensive properties, it is necessary to make further scientific progress in the development of novel quaternary ammonium monomers and in the optimization of the formulation of dental resins.

## Figures and Tables

**Table 1:** Antibacterial properties of representative quaternary ammonium monomers.

Quaternary Ammonium Monomer	Type *	Methods of Studying the Antimicrobial Activity	Resin-Based Dental Material	Antibacterial Properties
MDPB	1	MIC, MBC	primer	MIC: 12.5 μg/mL and MBC: 25.0 μg/mL against *Actinomyces israelii*. MIC: 3.13 μg./mL and MBC: 6.25 μg/mL against *Actinomyces gerensceriae*. MIC: 3.14–25.0 μg/mL and MBC: 6.25–50.0 μg/mL against *Actinomyces naeslundii*. MIC: 6.25 μg/mL and MBC: 12.5 μg/mL against *Actinomyces odontolyticus*. MIC: 3.13 μg/mL and MBC: 62.5 μg/mL against *Bifidobacterium bifidum*. MIC: 16.7 μg/mL and MBC: 31.3 μg/mL against *Streptococcus gordonii*. MIC: 25.0 μg/mL and MBC: 31.3 μg/mL against *Streptococcus mitis*. MIC: 12.5 μg/mL and MBC: 50.0 μg/mL against *S. mutans*. MIC: 12.5 μg/mL and MBC: 25.0 μg/mL against *Streptococcus oralis* [26]. The primer containing 5% MDPB exhibited strong antibacterial activity against *S. mutans*, *L. casei*, and *A. naeslundii* [27].
DMEA-BC; DMAE-CB; DMAE-m-CBC	1	MIC	adhesives	DMAE-CB exhibited the lowest MIC: 1.2~4.8 μg/mL. The killing rate against *S. mutans* of DMAE-CB reached 100% after 10 min [28]. The adhesives contained 3% DMAE-CB and showed the best antibacterial activity, and this effect lasted for at least 180 days. The cured specimens’ surface inhibited the growth of *S. mutans* [29].
QAM-n	1	CFU counts	resin	C18- and C16-containing copolymers had inhibition effects on both young and mature biofilms [30].
DHMAI	1	CFU counts, scanning electron microscopy (SEM)	resin composites	The composite with 7.5% DHMAI achieved a reduction of ∼98% in CFU and ∼50% of metabolic activity [31].
DMAPM (CL = 3); DMAHM (CL = 6); DMANM (CL = 9); DMADDM (CL = 12); DMAHDM (CL = 16); DMAODM (CL = 18)	1	CFU counts	bonding agent	Incorporating QAMs into Scotchbond multi-purpose (SBMP) reduced bacteria early attachment. Microcosm biofilm CFU for CL = 16 was 4 log lower than SBMP control [32]. Increasing the charge density reduced bacteria attachment and decreased biofilm CFU by 4 log [32]. The CFU of 3 wt% DMAHDM-containing composite resins was reduced by 2–3 logs compared to the control groups, and significantly inhibited early attachment of bacteria at 4 h and early biofilm formation at 24 h [33].
MAE-DB; MAE-HB	2	MBC	resin composites	Both MAE-DB and MAE-HB demonstrated strong antibacterial activity against eight strains of oral bacteria, with MBC values ranging from 12.2 to 24.4 μg/mL and 6.2 to 48.8 μg/mL, respectively [34]. The resultant polymers hold that the antibacterial activity lasted up to 180 days [35].
UDMQAs	2	CFU counts, agar diffusion test	resin composites	All UDMQA-based polymers showed antibacterial activity [36]. As for the UDMQA-12-containing polymers, they displayed a significant antibacterial activity at a concentration of 30% or more [37].
IMQs	2	CFU counts	resin composites	All dental resin composites with 10 wt% or more IMQs showed antibacterial activity [38]. The polymers containing IMQ-16 displayed the best comprehensive performance, and the ideal concentration of IMQ-16 ranged 5–10 wt% [38].
IDMA-1; IDMA-2	2	CFU counts, SEM	resin composites	Polymers containing 10% or more IDMA-1 can significantly reduce bacterial growth without compromising mammalian cell viability [39].
QANMA	2	bacteria colony counting,bacteria LIVE/DEADstaining	resin system	The dental resins containing QANMA showed significantly antibacterial properties, particularly with 10% and 20% QANMA [40].
Gemini quaternary ammonium salts (QASs)	3	MIC, MBC, CFU counts	dental restoration resin system	Six of these monomers were incorporated into dental restoration resin system. Among these, four monomers showed superior antibacterial activity against *S.* aureus with MIC values below 10 μg/mL, compared to conventional antibacterial agents [41]. The symmetric Gemini QAS monomers exhibited weaker antibacterial properties than the asymmetric Gemini QAS [41].
QAUDMAs	3	CFU counts, Inhibition Zone, bacteria cell proliferation	dental composites	No adhered *S. aureus* were observed on the QA14:TEG copolymer surface. The remaining decreases in the number of adhered *S. aureus* on other QA:TEG copolymers surface were lower than that of BG:TEG (Bis-GMA:TEGDMA) reference sample, from 35.57 to 86.68% [42]. *E. coli* were only observed on the surfaces of the QA16:TEG and QA18:TEG copolymers. No *E. coli* were observed on the surfaces of the remaining QA:TEG copolymers (from QA8:TEG to QA14:TEG) [42]. The pattern observed for the changes in bacteria number with the changing length of the N-alkyl substituent is consistent with the literature data [42]. All QA:TEG copolymers reduced the proliferation of *S. aureus*, while only copolymers containing QA8 to QA14 reduced the proliferation of *E. coli* [42].
bis-QAMs	3	CFU counts	resin composites	biQAMA-12 showed an exceeding 99% antibacterial efficiency and had no negative effect on the cytotoxicity of DRC [43].
EBet; BBet; HBet	3	CFU counts, SEM	resin composites	The experimental resins had stronger antibacterial activity with a more than 99.9% antibacterial efficiency against *S. mutans*, while the 1BBet4B5T resin had the best performances [44]. Bis-QADM-Bet inhibited the expression of all targeted genes (*gtfB*, *gtfC*, and *gtfD*) of *S. mutans*, disrupted the biofilm metabolism, and ultimately led to bacterial death [44].
DDMAI	4	CFU counts, MIC, SEM	resin composites	The MIC value of DDMAI against *S. mutans* was about 6.25 μg/mL [45]. DDMAI inhibited the formation of young *S. mutans* biofilm, but no inhibitory effect on 24 h mature biofilm [46]. 5 wt% DDMAI contained polymer having inhibitory effect on 6 h young and 24 h mature biofilm formation [46]. The polymers containing 15% or more 20% DDMAI strongly inhibited biofilm formation, and for 25% DDMAI, no viable cells or visible biofilm could be detected on the material surfaces [47].
QADMAIs	4	CFU counts	resin composites	All of QADMAIs could endow polymers with good inhibitory effect on biofilm formation [48].
IPhene	4	CFU counts	resin composites	Polymers with 30 wt% or more of IPhene had antibacterial activity [49].
TAs (DMAEM and HMAEM)	5	antibiofilm effect	resin adhesive	DMAEM and HMAEM have antibacterial activity only in an acidic environment [50]. The resin adhesive, TA@RAs showed a long-term antibacterial activity and the ability to prevent secondary dental caries [50].

* 1. Mono-methacrylate quaternary ammonium monomer; 2. di-methacrylate quaternary ammonium monomer; 3. bi-quaternary ammonium monomer; 4. radio-opaque and antibacterial bifunctional agent; 5. smart monomer.

## Data Availability

Not applicable.
